# *Telmatometropsis fredyi* gen. nov., sp. nov.: a new water strider from the Colombian Pacific region (Insecta, Hemiptera, Gerridae)

**DOI:** 10.3897/zookeys.1043.58548

**Published:** 2021-06-11

**Authors:** Silvia P. Mondragón-F., Irina Morales, Felipe F. F. Moreira

**Affiliations:** 1 Laboratorio de Entomología, Universidad Pedagógica y Tecnológica de Colombia, Avenida Central del Norte 39-155, Tunja, BY, Colombia Universidad Pedagógica y Tecnológica de Colombia Tunja Colombia; 2 Laboratório de Biodiversidade Entomológica, Instituto Oswaldo Cruz, Fundação Oswaldo Cruz, Av. Brasil, 4365, Pavilhão Mourisco, sala 214. Manguinhos, Rio de Janeiro, RJ, Brazil Instituto Oswaldo Cruz, Fundação Oswaldo Cruz Rio de Janeiro Brazil

**Keywords:** Aquatic insects, Gerromorpha, Neotropical Region, taxonomy

## Abstract

A new genus of Gerridae (Insecta, Hemiptera, Heteroptera) in the subfamily Trepobatinae, *Telmatometropsis***gen. nov.**, with a single included species, *T. fredyi***sp**. **nov.**, is described from the Colombian Pacific region. Representatives of the new genus were collected in mangrove lagoons of Buenaventura Bay, Valle del Cauca Department. The new genus can be diagnosed by the relative proportions of the antennomeres, the shape of the male fore tarsus, and by the black markings on the head, thorax and abdomen.

## Introduction

Gerridae comprises over 750 described species in more than sixty genera and eight subfamilies, of which almost 150 species have been recorded from the Neotropical Region (Polhemus and Polhemus 2008). Representatives of the family are semiaquatic bugs that spend almost their entire lives skating on the water surface of lentic and lotic environments (Andersen 1982; Schuh and Slater 1995). Most gerrids live in freshwater, but a handful inhabit the open ocean (Andersen 1998) and others occupy estuarine brackish waters (Cheng 1976). According to Molano-Rendón and Morales (2017), the subfamilies Halobatinae, Rhagadotarsinae and Trepobatinae have species with marine habits. For example, some species of the genus *Rheumatobates* Bergroth, 1892 inhabit estuarine brackish waters on the Caribbean and Pacific coasts of Central and South America, and species of *Halobates* Eschscholtz, 1822 are almost exclusively marine, with five species living on the open ocean (Andersen and Cheng 2004; Cheng 2006).

The subfamily Trepobatinae is represented in the Neotropical Region by the genera *Halobatopsis* Bianchi, 1896; *Lathriobatoides* Polhemus, 2004; *Metrobates* Uhler, 1871; *Ovatametra* Kenaga, 1942; *Telmatometra* Bergroth, 1908; *Telmatometroides* Polhemus, 1991; *Trepobates* Uhler, 1894; and *Trepobatoides* Hungerford & Matsuda, 1958 (Polhemus and Polhemus 2002). *Telmatometroides* differs from the others by antennomere III shorter than two times the length of antennomere II; interocular space with a dark longitudinal stripe; extensive black markings on posterior part of mesosternum; row of five or six short, stout, black spinose setae on hind tarsomere I; and mid femur shorter than mid tibia and hind femur (Polhemus 1991; Moreira et al. 2018). Currently, *Telmatometroides* is a monospecific genus, including only *T. rozeboomi* (Drake & Harris, 1937), which is recorded from Costa Rica to Ecuador (Pacheco-Chaves et al. 2018).

We recently noticed that some of the specimens deposited in the collection of Universidad Pedagógica y Tecnológica de Colombia, Tunja, Colombia, and identified as *T. rozeboomi* did not agree with the features mentioned in the description of this species, especially regarding the relative proportions of the antennomeres and the disposition of black markings on the head and body. A more detailed examination also showed a modification on the male fore tarsus that is not reported for any Neotropical genus of Trepobatinae, thus revealing an undescribed genus and species from the Colombian Pacific region that are herein described.

## Methods

Type specimens have been deposited in the following collections: Colección de Insectos, Museo de Historia Natural “Luis Gonzalo Andrade”, Universidad Pedagógica y Tecnológica de Colombia, Tunja, Colombia (**UPTC**); and Coleção Entomológica do Instituto Oswaldo Cruz, Fundação Oswaldo Cruz, Rio de Janeiro, Brazil (**CEIOC**). For comparison with the new genus, we examined photographs of the holotype of *Telmatometroides rozeboomi*, which is deposited in the National Museum of Natural History, Smithsonian Institution, Washington, D.C., United States of America (**NMNH**). Micrographs of the new species were taken using a Zeiss EVO MA10 scanning electron microscope. A Leica S9I stereo microscope with integrated camera was used to obtain the photographs and measurements of 10 specimens of each sex (including the holotype). All measurements are given in millimeters. They are abbreviated as follows in Tables 2 and 3: body length (BL), body width (BW), head width through eyes (HW), lengths of antennomeres I–IV (ANT I, ANT II, ANT III, ANT IV), pronotum length on midline (PL), abdomen length on midline (AL), femoral length (FEM), tibial length (TIB), and lengths of tarsomeres I–II (TAR I, TAR II). The software QGIS (3.4) was used to generate the geographic distribution map.

## Results

### Telmatometropsis gen. nov.

Taxon classificationAnimaliaHemipteraGerridae

ED5677CF-6880-564D-A138-312EBDBDF5FF

http://zoobank.org/3C030D62-5C05-466A-AD9A-1FA9E5AA241A

Figs 1 , 2 , 3 , 4 , 5 , 6 , 7 , 8

#### Type species.

*Telmatometropsis fredyi* Mondragón-F., Morales & Moreira sp. nov., by present designation and monotypy.

**Figure 1.**
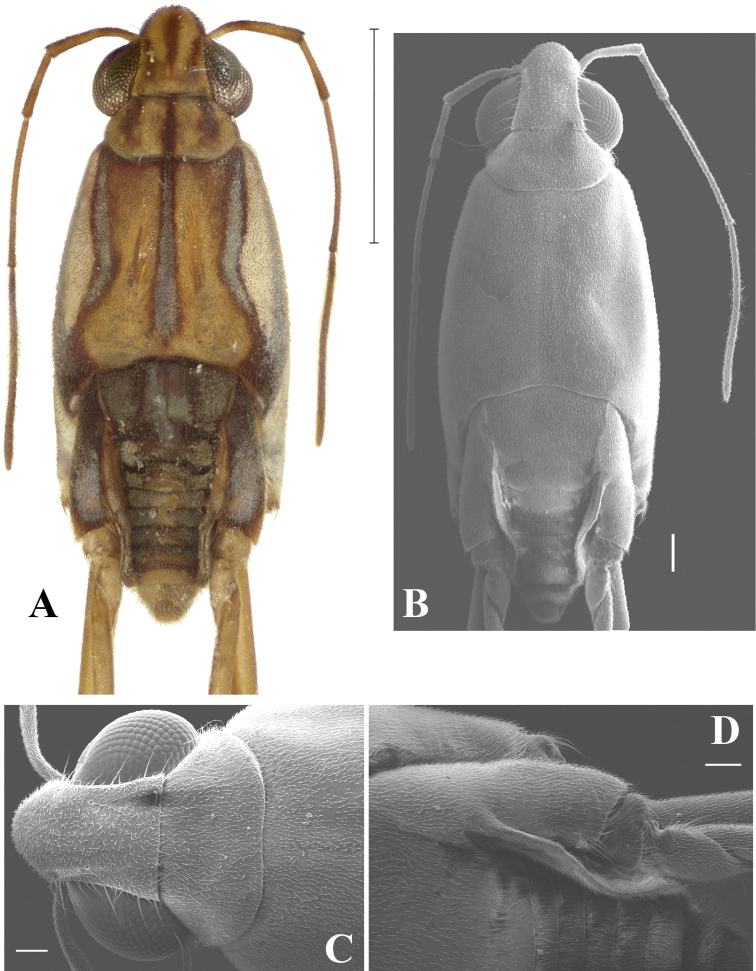
*Telmatometropsis fredyi* gen. nov, sp. nov., male **A** dorsal view **B–D** Scanning electron micrographs **B** dorsal view **C** head, pronotum and anterior portion of mesonotum, dorsal view **D** apex of thorax and base of abdomen, dorsal view. Scale bars: 1 mm (**A**); 400 μm (**B**); 100 μm (**C, D**).

**Figure 2.**
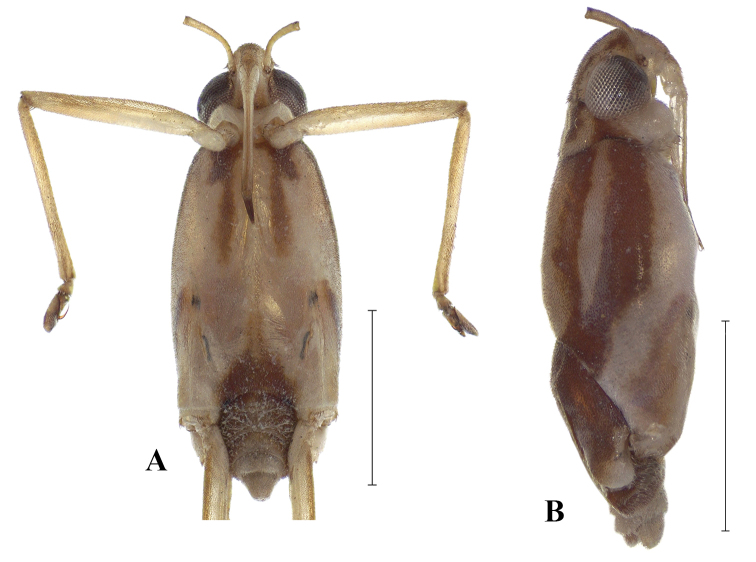
*Telmatometropsis fredyi* gen. nov, sp. nov., male **A** ventral view **B** lateral view. Scale bars: 1 mm.

#### Diagnosis.

The new genus is similar to *Telmatometroides* (Fig. 9), sharing with it the long ocular setae, the median and lateral longitudinal black stripes on the mesonotum, male abdominal tergum VIII with a central notch on the posterior margin (stronger on *T. rozeboomi*), mid tarsomere I with a few bristles at the base, the hind femur with five dorsal trichobothria, the male abdominal laterotergites with patches of light setae, and the occupation of estuarine brackish waters. *Telmatometropsis* gen. nov. differs from *Telmatometroides* and all other genera of Neotropical Trepobatinae by the modified fore tarsomere II of the male, which is strongly curved in lateral view, flattened laterally, and apically bifid, with a shorter and a longer portion. The relative proportions of the antennomeres are also unique to the new genus, with article III clearly longer than article I, more than twice as long as article II, but shorter than article IV. Further comparison with other genera of Neotropical Trepobatinae is given in Table 1 and in an updated key to Neotropical Trepobatinae genera provided below.

**Table 1.** Comparison of *Telmatometropsis* gen. nov. with other genera of Neotropical Trepobatinae. Data on other genera were obtained from Drake and Harris (1932), Kenaga (1941, 1942), Hungerford and Matsuda (1958), Andersen (1982), Polhemus (1991), Nieser (1993), Nieser and Melo (1999), and Aristizábal-García (2017), but not exhaustively concerning measurements.

	Habitat	Ground color	Interocular marks	Mesonotal marks	BL	ANT III/ANT I	ANT III/ANT II	ANT III/ANT IV
*** Telmatometropsis ***	Marine	Yellow	Present	Median+lateral	2.90–3.90	1.25–1.85	2.05–2.40	0.72–0.90
*** Halobatopsis ***	Freshwater	Yellow/brown	Present/absent	Median+lateral/absent	3.40–4.60	0.68–0.77	1.33–1.42	0.79–1.12
*** Lathriobatoides ***	Freshwater	Yellow/brown	Absent	Absent	2.60–3.20	1.10–1.20	1.70	1.10–1.20
*** Metrobates ***	Freshwater	Black	Present	Median/median+lateral	3.00–5.00	0.22–0.30	0.73–0.84	0.67–1.29
*** Ovatametra ***	Freshwater	Yellow/brown	Present	Median+lateral	2.00–3.10	0.70	1.20	0.64–0.71
*** Telmatometra ***	Freshwater	Yellow/brown	Present/absent	Median+lateral/lateral	3.30–5.50	1.20–1.40	2.20–2.40	1.00–1.42
*** Telmatometroides ***	Marine	Yellow	Present	Median+lateral	3.15–3.70	1.10–1.20	1.40–1.50	0.94
*** Trepobates ***	Freshwater/marine	Yellow/brown/black	Present	Variable	3.00–5.50	0.70	1.10–1.20	0.84–0.97
*** Trepobatoides ***	Freshwater	Yellow/brown	Present	Median+lateral	3.57–4.45	0.40	1.20	0.68–0.70

#### Description.

**Figure 3.**
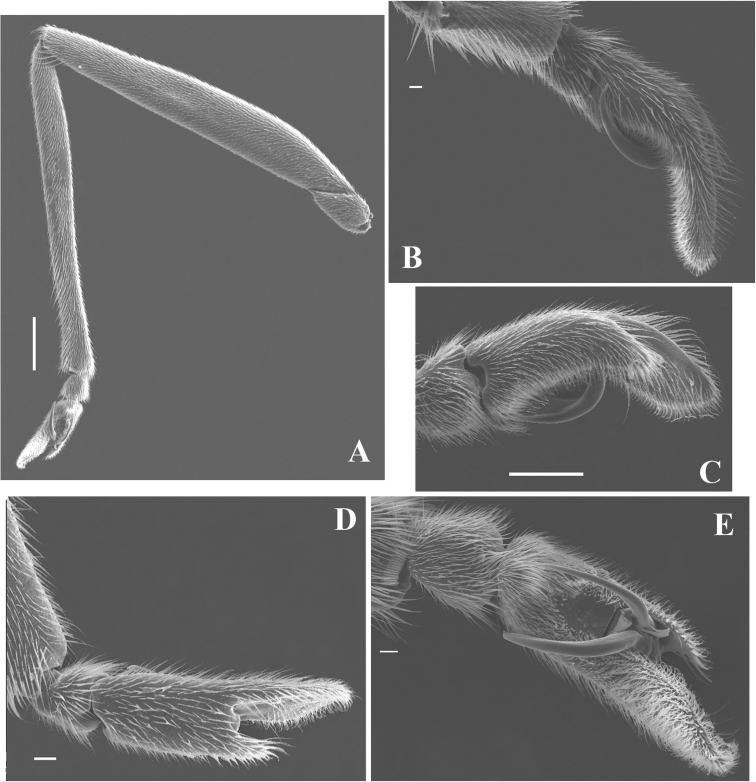
*Telmatometropsis fredyi* gen. nov, sp. nov., male, scanning electron micrographs **A** fore leg, ventral view **B–E** fore tarsus **B** external lateral view **C** internal lateral view **D** dorsal view **E** ventral view. Scale bars: 200 μm (**A**); 300 μm (**B**); 20 μm (**C**); 60 μm (**D**); 40 μm (**E**).

***Measurements*.** Male body length 2.90–3.21, width (across suture between meso- and metanotum) 1.07–1.21; female body length 3.30–3.91, width 1.44–1.52. ***Color*.** Ground color of body pale yellow with extensive black and silvery markings dorsally, legs largely pale yellow (Fig. 1A). ***Structural characteristics*.** Eyes elongate, with a pair of long ocular setae (Fig. 1B). Head with four pairs of trichobothria (Fig. 1C). Antenna shorter than body length; antennomere I thickest, curved laterally at base, longer than antennomere II; antennomere II shortest, thicker than antennomeres III and IV; antennomere III longer than antennomere I; antennomere IV longest (Fig. 1A, B). Labium long, extending to mesosternum. Pronotum short, trapezoid (Fig. 1C). Mesonotum about three times as long as pronotum, posterior margin slightly concave (Fig. 1A, B). Fore femur subequal in length to fore tibia, slightly curved at the base in dorsal view; fore tibia with apicolateral row of short, distinctive setae (Fig. 3A); fore tarsus covered with short yellow setae; fore tarsomere I about one third the length of fore tarsomere II; male fore tarsomere II strongly curved in lateral view, flattened laterally, apically bifid with a shorter and a longer portion; claws directed mesally (Fig. 3A–E). Mid femur about two-thirds the length of mid tibia; mid tibia less than twice the length of mid tarsus, about as long as medial length of body from anterior margin of pronotum to apex of abdomen, occasionally almost as long as body; mid tarsus shorter than mid femur, article I subequal to article II or a little longer. Hind femur longer than mid femur; hind tibia about two-thirds the length of hind femur, densely covered with setae; hind tarsus about half the length of hind tibia, article I longer than article II.

#### Etymology.

The generic name refers to its resemblance to the genus *Telmatometroides*.

### Telmatometropsis fredyi sp. nov.

Taxon classificationAnimaliaHemipteraGerridae

CED578D4-884A-5F01-BE21-8CCE0CB0BA68

http://zoobank.org/707ddb44-4b8c-493b-9372-2efd98ffcf0c

Figs 1 , 2 , 3 , 4 , 5 , 6 , 7 , 8 , Tables 2 , 3

#### Description.

**Figure 4.**
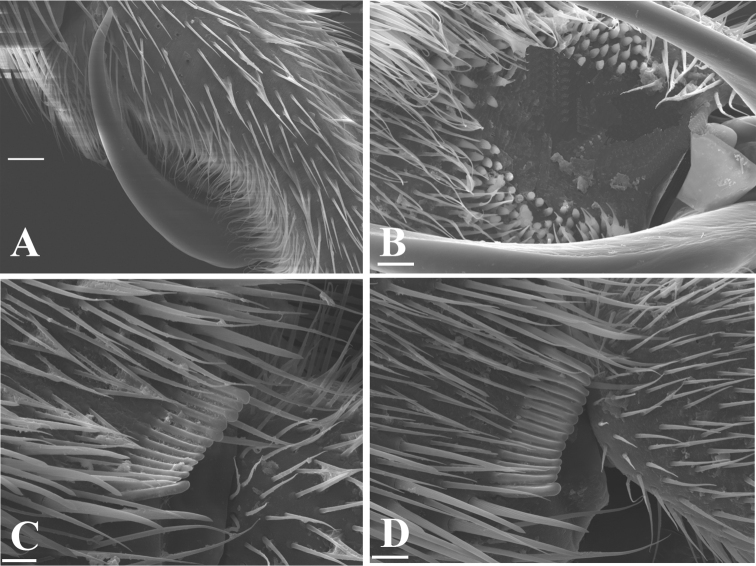
*Telmatometropsis fredyi* gen. nov, sp. nov., scanning electron micrographs **A–C** male **A** fore pretarsal claw, lateral view **B** fore tarsomere II, area with cuticular pegs adjacent to pretarsal claw insertion, ventral view **C** apex of fore tibia with grooming structures, ventral view **D** female, apex of fore tibia with grooming structures, ventral view. Scale bars: 20 μm (**A**); 10 μm (**B, C, D**).

**Figure 5.**
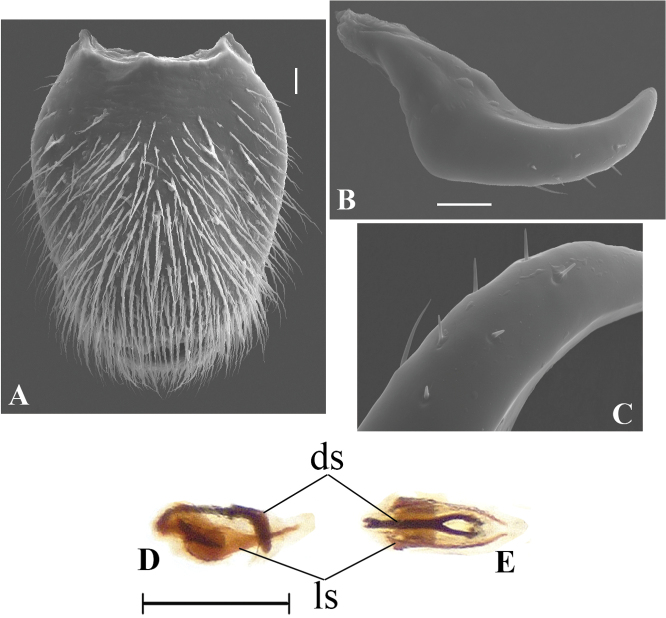
*Telmatometropsis fredyi* gen. nov, sp. nov., male, scanning electron micrographs **A** proctiger, dorsal view **B** paramere, lateral view **C** detail of paramere setiferation **D–E** sclerites **D** lateral view **E** dorsal view. Abbreviations: sclerite (ls), dorsal sclerite (ds). Scale bars: 40 μm (**A**); 20 μm (**B**); 0.25 mm (**D–E**).

**Male.** [For measurements see Table 2.] ***Color*.** Ground color yellow. Head dorsally pale yellow, with three longitudinal brown stripes between eyes; stripes connected posteriorly (Fig. 1A). Venter of head light brown (Fig. 2A). Eye golden brown. Antenna yellowish-brown to brown. Labial articles I–III light brown with brown longitudinal stripe; article IV dark brown. Pronotum pale yellow, with a dark brown median stripe throughout length and laterally with a pair of dark brown longitudinal stripes reaching slightly beyond middle of length. Mesonotum pale yellow, covered with silvery pilosity, with brown spots on anterior margin, a dark brown median stripe almost reaching posterior margin, and a pair of dark brown longitudinal stripes laterally; lateral stripes posteriorly narrowed and connected to mesopleural stripes (Fig. 2B). Metanotum velvety, dark brown with central subtriangular spot of dense silvery pubescence and two lateral spots of silvery pubescence. Mesopleura covered with silvery pubescence, with a longitudinal dark brown stripe on its ventralmost portion; stripe posteriorly connected to mesonotal stripe. Meso- and metacetabula with pruinose patches. Prosternum pale yellow; mesosternum pale yellow with two longitudinal brown spots (Fig. 2A); metasternum with posterior subtriangular dark brown spot (Fig. 2A). Abdominal mediotergite I dark brown, with silvery pubescence on posterolateral corners; mediotergite II dark brown, with central yellow spot posteriorly and silvery pubescence on posterolateral corners; mediotergites III–VI dark brown, covered with pruinose layer, with central yellow spots varying in size; mediotergites VII–VIII pale yellow, covered with abundant pubescence of same color; mediotergite VII dark brown on anterior margin. Abdominal laterotergites yellow, with patches of light setae. Abdomen laterally dark brown. Foreleg: coxa pale yellow; trochanter pale yellow, with brown lateral fringe; femur dorsally brown, ventrally pale yellow, mesal margin larerally yellow, with longitudinal white line; tibia brown; tarsus dark brown. Mid leg: coxa pale yellow; trochanter brown; femur dorsally brown, ventrally pale yellow; tibia with basal half brown and apical half dark brown; tarsus dark brown. Hind leg: coxa pale yellow; trochanter pale yellow; femur dorsally brown, ventrally pale yellow with dark brown apex; tibia and tarsus dark brown. ***Structure*.** Head with frons rounded. Antennomere I curved laterally; antennomere II shortest; antennomere IV longest (Fig. 1A, B). Fore leg: femur widened in basal half, ventrally flattened (Fig. 3A), with ventrolateral row of 4–7 small bristles; apex of tibia with long black bristles laterally and grooming structures (Fig. 4C); tarsomere I 1/4 of tarsomere II length; tarsomere II strongly curved in lateral view, flattened laterally, and apically bifid, with a shorter and a longer portion, with cuticular subconical pegs (Fig. 4B); claws long, strongly curved back (Figs 3B, C, E, 4A). Mid tarsomere I with few bristles at base. Hind femur with five dorsal trichobothria. Abdominal laterotergites elevated at approximately 45° (Fig. 1A, B, D). Abdominal segment VIII dorsally with a small central notch on posterior margin (Fig. 1A, B). Paramere with apex curved up and rows of bristles close to apex (Fig. 5B, C); proctiger oval, covered with setae (Fig. 5A); sclerites as in Fig. 5 D–E.

**Table 2.** Measurements of male morphological structures of *Telmatometropsis fredyi* sp. nov.

Structure	Male 1	Male 2	Male 3	Male 4	Male 5	Male 6	Male 7	Male 8	Male 9	Male 10	Maximum	Minimum	Average
**BL**	3.17	3.01	3.20	3.02	3.04	2.90	3.05	3.21	3.18	2.90	3.21	2.90	3.07
**BW**	1.13	1.09	1.11	1.07	1.21	1.13	1.13	1.19	1.12	1.12	1.21	1.07	1.13
**HW**	0.28	0.28	0.32	0.31	0.32	0.29	0.31	0.35	0.32	0.31	0.35	0.28	0.31
**ANT I**	0.48	0.40	0.47	0.45	0.40	0.44	0.48	0.44	0.47	0.47	0.48	0.40	0.45
**ANT II**	0.29	0.31	0.35	0.30	0.32	0.33	0.34	0.32	0.32	0.31	0.35	0.29	0.32
**ANT III**	0.70	0.71	0.75	0.71	0.74	0.68	0.72	0.68	0.71	0.66	0.66	0.75	0.71
**ANT IV**	0.85	0.80	0.95	0.95	0.97	0.94	0.91	0.84	0.85	0.87	0.97	0.80	0.89
**PL**	0.30	0.27	0.32	0.28	0.31	0.30	0.32	0.30	0.31	0.31	0.32	0.27	0.30
**AL**	1.23	1.10	1.10	1.03	1.03	1.16	1.21	1.23	1.27	1.12	1.27	1.03	1.15
**Fore leg**													
**FEM**	1.10	1.13	1.11	1.02	1.07	1.16	1.14	1.14	1.16	1.16	1.16	1.02	1.12
**TIB**	1.10	1.19	1.14	1.12	1.12	1.15	1.16	1.12	1.16	1.11	1.19	1.10	1.14
**TAR I**	0.10	0.06	0.07	0.07	0.10	0.08	0.08	0.09	0.09	0.09	0.10	0.06	0.08
**TAR II**	0.27	0.32	0.31	0.31	0.26	0.31	0.29	0.28	0.31	0.34	0.34	0.26	0.30
**Mid leg**													
**FEM**	1.82	1.83	1.77	1.75	1.79	1.82	1.86	1.74	1.86	1.75	1.86	1.74	1.80
**TIB**	2.64	2.64	2.61	2.46	2.69	2.55	2.66	2.61	2.62	2.62	2.69	2.46	2.61
**TAR I**	0.93	0.87	0.87	0.63	0.88	0.81	0.87	0.80	0.82	0.88	0.93	0.63	0.84
**TAR II**	0.68	0.71	0.73	0.52	0.79	0.73	0.70	0.73	0.50	0.68	0.79	0.50	0.68
**Hind leg**													
**FEM**	2.27	2.19	2.21	2.19	2.22	2.22	2.24	2.25	2.10	2.23	2.27	2.10	2.21
**TIB**	0.93	0.92	0.93	0.89	0.91	0.81	0.92	0.88	0.92	0.92	0.93	0.81	0.90
**TAR I**	0.22	0.21	0.22	0.21	0.22	0.19	0.21	0.21	0.22	0.21	0.22	0.19	0.21
**TAR II**	0.27	0.27	0.27	0.26	0.28	0.25	0.26	0.26	0.26	0.27	0.28	0.25	0.27

**Figure 6.**
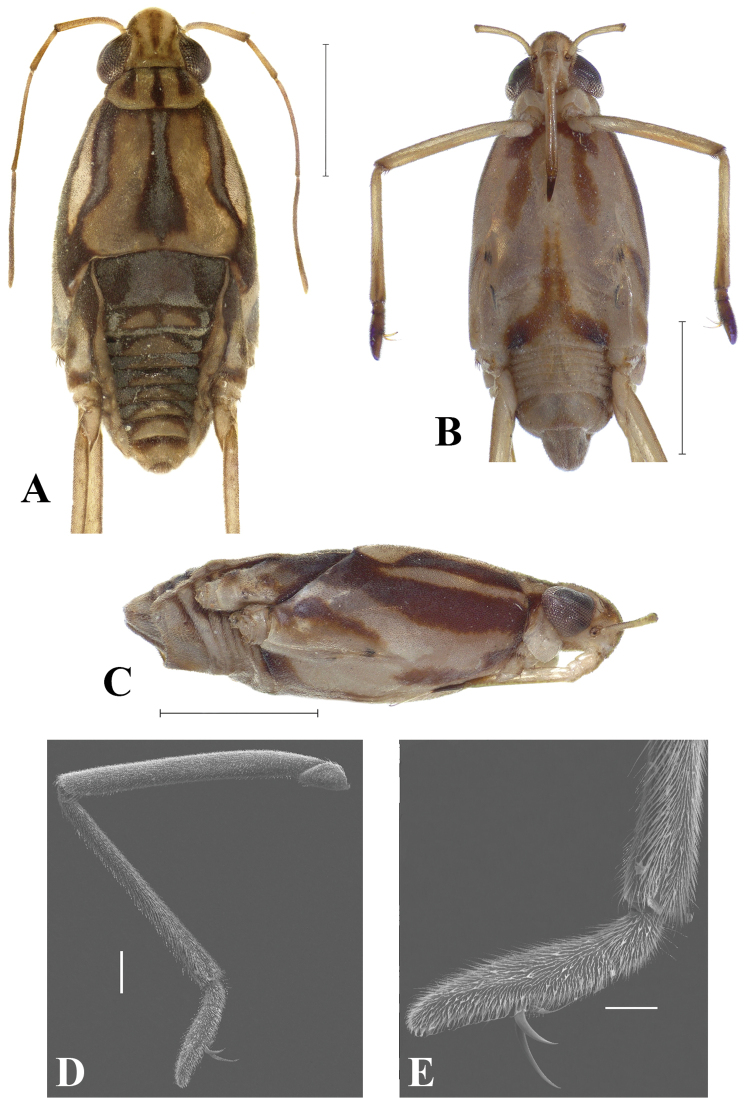
*Telmatometropsis fredyi* gen. nov, sp. nov., female **A** dorsal view **B** ventral view **C** lateral view **D–E** scanning electron micrographs **D** fore leg, ventral view **E** apex of fore tibia and fore tarsus, ventral view. Scale bars: 1 mm (**A, B, C**); 200 μm (**D**); 100 μm (**E**).

**Female.** [For measurements see Table 3.] Similar in color and structure to male, but larger and more robust (Fig. 6A–C). Central spot on metanotum quadrate (Fig. 6A). Spot on metasternum inverted “T”-shaped (Fig. 6B). Abdominal mediotergites dark brown, with central yellow spots; mediotergites II–IX with pruinose patches laterally (Fig. 6C); laterotergites without patches of light setae (Fig. 6A). Fore leg: femur slightly curved at the base (Fig. 6D), with 3–7 bristles; tibia with grooming structures (Fig. 4D); tarsomere II cylindrical, flattened laterally (Fig. 6E). Abdominal laterotergites elevated by almost 90° (Fig. 6A).

**Table 3.** Measurements of female morphological structures of *Telmatometropsis fredyi* sp. nov.

Structure	Female 1	Female 2	Female 3	Female 4	Female 5	Female 6	Female 7	Female 8	Female 9	Female 10	Maximum	Minimum	Average
**BL**	3.30	3.62	3.65	3.54	3.56	3.46	3.47	3.91	3.85	3.42	3.91	3.30	3.58
**BW**	1.50	1.50	1.51	1.52	1.51	1.47	1.44	1.50	1.50	1.46	1.52	1.44	1.49
**HW**	0.34	0.34	0.36	0.35	0.35	0.36	0.37	0.36	0.36	0.36	0.37	0.34	0.36
**ANT I**	0.49	0.55	0.53	0.54	0.53	0.55	0.54	0.54	0.55	0.51	0.55	0.49	0.53
**ANT II**	0.35	0.34	0.36	0.35	0.34	0.37	0.33	0.36	0.36	0.34	0.37	0.33	0.35
**ANT III**	0.74	0.71	0.74	0.73	0.78	0.81	0.68	0.83	0.79	0.73	0.83	0.68	0.75
**ANT IV**	0.91	0.79	0.95	1.02	0.97	0.95	0.91	1.00	0.93	0.91	1.02	0.79	0.93
**PL**	0.29	0.32	0.33	0.30	0.31	0.33	0.34	0.34	0.32	0.30	0.34	0.29	0.32
**AL**	1.53	1.71	1.72	1.70	1.72	1.63	1.64	1.90	1.82	1.60	1.90	1.53	1.70
**Fore leg**													
**FEM**	1.12	1.08	1.17	1.13	1.18	1.19	1.14	1.14	1.14	1.18	1.19	1.08	1.15
**TIB**	1.04	1.07	1.10	1.07	1.09	1.08	1.09	1.10	1.13	1.10	1.13	1.04	1.09
**TAR I**	0.10	0.09	0.09	0.11	0.12	0.10	0.12	0.08	0.08	0.10	0.12	0.08	0.10
**TAR II**	0.34	0.43	0.43	0.37	0.40	0.41	0.40	0.40	0.43	0.39	0.43	0.34	0.40
**Mid leg**													
**FEM**	2.08	2.11	2.10	2.10	2.20	2.22	2.06	2.16	2.00	2.09	2.22	2.00	2.11
**TIB**	3.14	2.98	3.11	3.15	3.14	3.21	3.13	3.07	2.77	3.07	3.21	2.77	3.08
**TAR I**	1.09	0.96	1.07	1.07	1.00	1.09	1.02	1.02	0.96	1.06	1.09	0.96	1.03
**TAR II**	0.71	0.81	0.83	0.86	0.70	0.89	0.84	0.84	0.69	0.85	0.89	0.69	0.80
**Hind leg**													
**FEM**	2.60	2.52	2.63	2.46	2.67	2.63	2.63	2.67	2.67	2.61	2.67	2.46	2.61
**TIB**	1.15	1.11	1.14	1.17	1.15	1.13	1.12	1.12	1.05	1.14	1.17	1.05	1.13
**TAR I**	0.26	0.26	0.28	0.27	0.27	0.27	0.27	0.27	0.25	0.27	0.28	0.25	0.27
**TAR II**	0.33	0.32	0.33	0.34	0.33	0.35	0.34	0.35	0.33	0.31	0.35	0.31	0.33

#### Type material.

***Holotype*.** Colombia • apterous male; Valle del Cauca, Buenaventura, La Bocana, lagoon, 8.XI.2003; Molano & Camacho leg. (UPTC-In-00001). ***Paratypes*.** 1 apterous female; Valle del Cauca, Buenaventura, La Bocana, lagoon, 4.XI.2004, Molano & Morales leg. (UPTC-In-00002). 5 apterous males and 5 apterous females, same data as for holotype (UPTC-In-00003). 2 apterous males and 2 apterous females; Valle del Cauca, Buenaventura, La Bocana, lagoon, 4.XI.2004, Molano & Morales leg. (CEIOC 76834). 1 apterous female; Valle del Cauca, Buenaventura, Santa Clara, 17.V.2004, Camacho & Molano leg. (UPTC-In-00004). 4 apterous males and 12 apterous females; Valle del Cauca, Buenaventura, La Bocana, lagoon, 4.XI.2004, Molano & Morales leg. (UPTC-In-00208). 2 apterous males and 8 apterous females; Valle del Cauca, Buenaventura, La Bocana, lagoon, 8.XI.2003, Molano & Camacho leg. (UPTC In-00209). 1 apterous male and 1 apterous female; Valle del Cauca, Buenaventura, Punta Arenas, mangrove lagoons, 26.I.1986, M.R. Manzano leg. (UPTC-In-00210).

#### Etymology.

The new species is named in honor of Professor Fredy Molano, who made a great contribution to the knowledge of Gerromorpha from Colombia.

#### Habitat notes.

**Figure 7.**
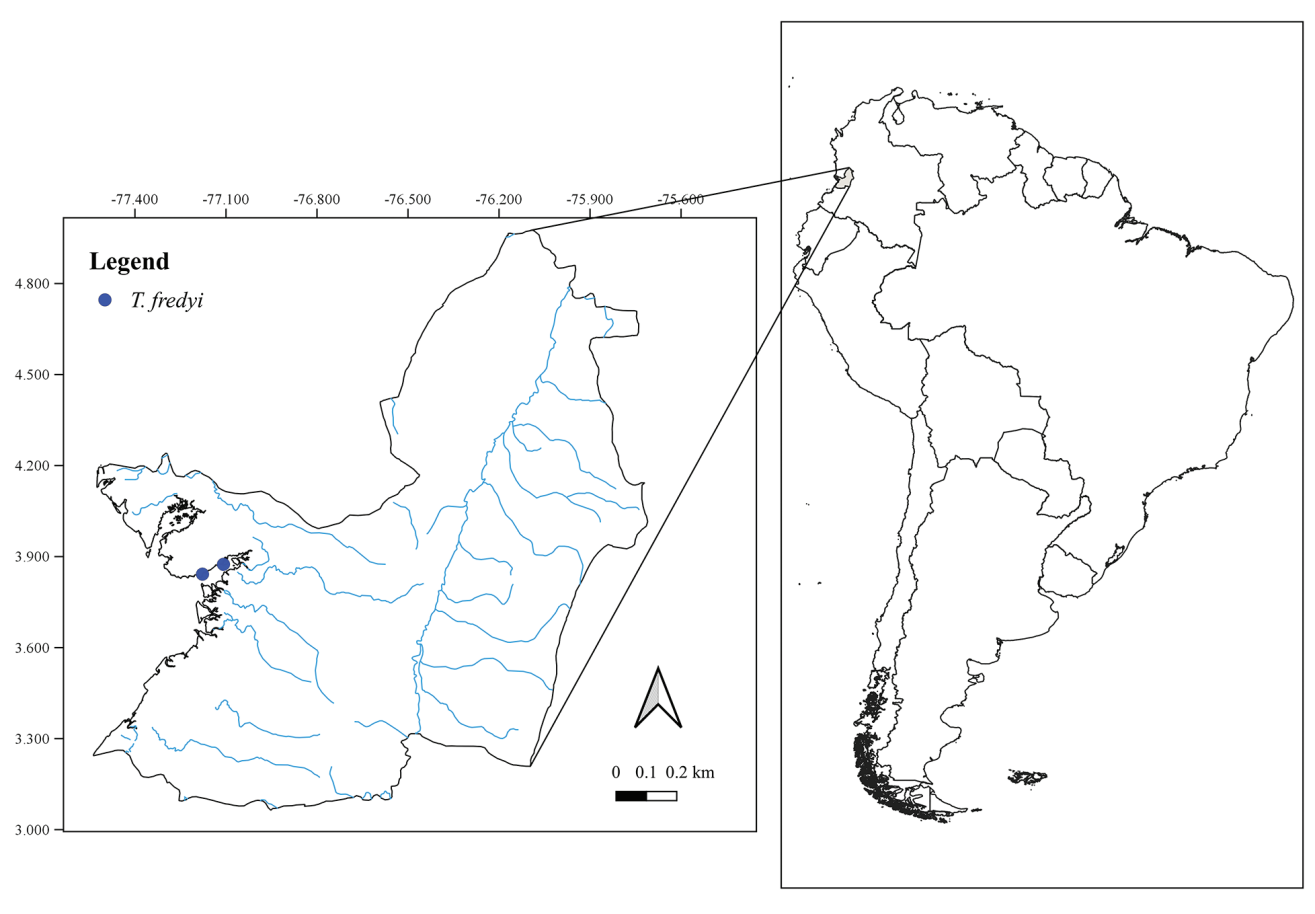
Geographical distribution of *Telmatometropsis fredyi* gen. nov, sp. nov.

**Figure 8.**
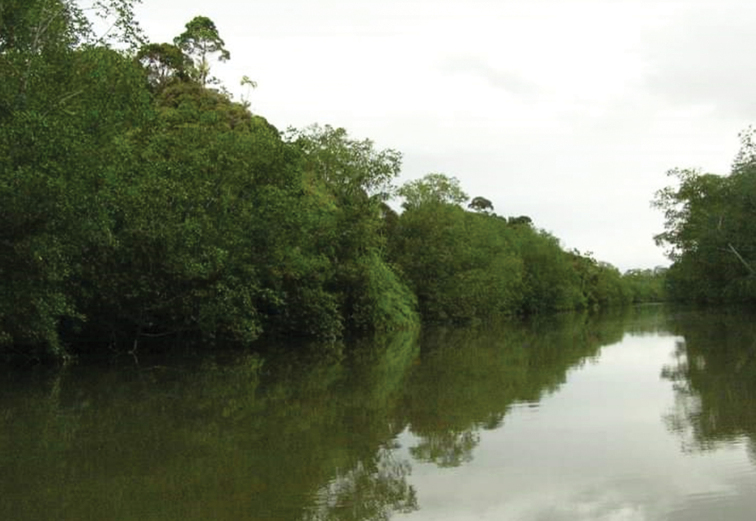
Type locality of *Telmatometropsis fredyi* sp. nov.; mangrove lagoons in Buenaventura Bay, Valle del Cauca, Colombia.

The species inhabits mangrove lagoons in Buenaventura Bay, Valle del Cauca Department, Pacific region of Colombia (Figs 7, 8).

**Figure 9.**
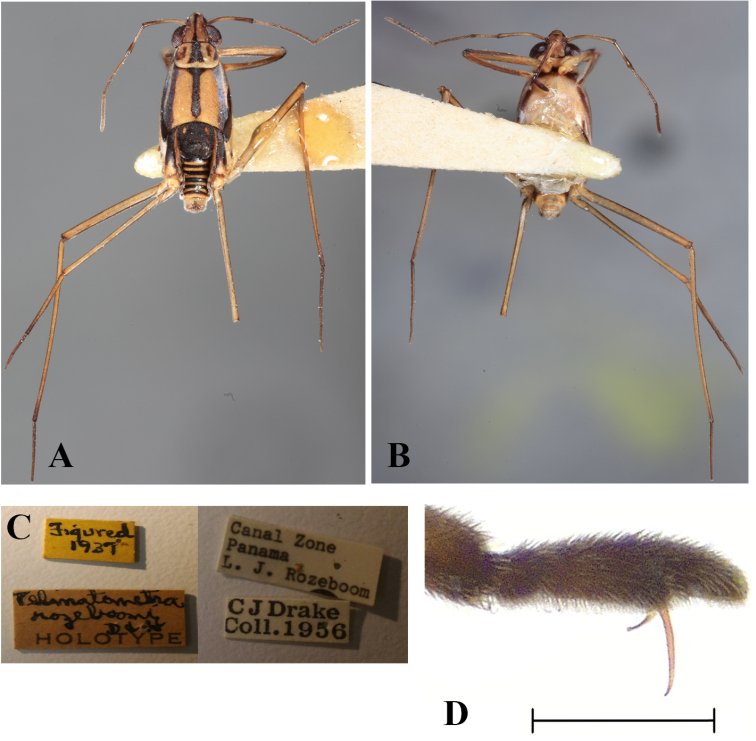
Apterous holotype male, *Telmatometroides rozeboomi* (Drake & Harris, 1937) **A** dorsal view **B** ventral view **C** labels **D** fore tarsus, internal lateral view. Scale bar: 0.25 mm.

### Key to the genera of Neotropical Trepobatinae

Modified from Moreira et al. 2018.

**Table:** 

1	Antennomere II longer than antennomere III; antennomeres II–III of male distally widened	*** Metrobates ***
–	Antennomere II subequal in length or shorter than antennomere III; antennomeres II–III of male not distally widened	**2**
2	Antennomere III 10–85% longer than antennomere I	**3**
–	Antennomere III 40–80% of length of antennomere I	**6**
3	Antennomere III shorter than two times the length of antennomere II	**4**
–	Antennomere III longer than two times the length of antennomere II	**5**
4	Interocular space with a dark longitudinal stripe	*** Telmatometroides ***
–	Interocular space without a dark longitudinal stripe	*** Lathriobatoides ***
5	Antennomere IV not the longest; male fore tarsus unmodified, cylindrical; freshwater habitats	*** Telmatometra ***
–	Antennomere IV the longest; male fore tarsus modified (Fig. 2); marine habitats	***Telmatometropsis* gen.nov.**
6	Antennomere I much longer than antennomeres II–III together	*** Trepobatoides ***
–	Antennomere I at most as long as antennomeres II–III together	**7**
7	Mid tibia distinctly shorter than length of body	*** Ovatametra ***
–	Middle tibia almost as long as or slightly longer than length of body	**8**
8	Eye in lateral view not extending beyond half of propleuron; hind tibia distinctly shorter than two times the length of hind tarsus	*** Trepobates ***
–	Eye in lateral view extending beyond half of propleuron; hind tibia longer than two times the length of hind tarsus	*** Halobatopsis ***

## Discussion

The municipality of Buenaventura has a monomodal precipitation regime, with a tendency to bimodality. The highest precipitation values occur between the months of September and October, while the lowest values are observed between February and March, with an average annual precipitation of 7400 mm and an average temperature of 25.9 °C. These very particular climatological characteristics generate a very humid and warm climate (Enriquez et al. 2014). Several freshwater bodies flow to Buenaventura Bay, such as the Dagua River, and the Aguadulce, Pichido, El Corral, and San Joaquín streams. These different estuaries with a large number of drains constitute a deltaic system in the area. The bay is geomorphologically characterized by the vegetated intertidal platforms, which correspond to muddy plains of fine sediments and abundant organic matter, where mainly mangrove-type vegetation grows (*Avicennia germinans* (L.) Stearn (Lamiales: Acanthaceae), *Laguncularia racemosa* (L.) Gaertn. (Myrtales: Combretaceae), *Rhizophora mangle* L., and *R. harrisonii* Leechm. (Malpighiales: Rhizophoraceae)) (Álvarez et al. 2016). It is in such places that the type specimens of *Telmatometropsis fredyi* sp. nov. were collected.

The new genus herein described has a unique feature, the strongly modified male fore tarsomere II, which in other Neotropical trepobatines is elongated and cylindrical. The distribution of *Telmatometropsis* gen. nov. partially overlaps with that of *Telmatometroides*. However, these two genera apparently do not share the same microhabitats, since they have not been collected together. The new genus probably also occurs in the departments of Chocó and Nariño, both in the Colombian Pacific region and where several mangroves are found.

## Supplementary Material

XML Treatment for Telmatometropsis

XML Treatment for Telmatometropsis fredyi
